# The continuing evolution of a cancer prevention, screening, and survivorship ECHO: A second year of implementation

**DOI:** 10.1002/cam4.5441

**Published:** 2022-12-12

**Authors:** Mary Ann Etling, Terry A. Vik, Andrea D. Janota, Kaley L. Liang, Caroline L. Kryder‐Reid, Mary Robertson, Caitlin Scanlon, Anyé Carson, Jon Agley, Tyler S. Severance

**Affiliations:** ^1^ Indiana University School of Medicine Indianapolis Indiana USA; ^2^ Indiana University Richard M. Fairbanks School of Public Health Indianapolis Indiana USA; ^3^ Riley Hospital for Children Indianapolis Indiana USA; ^4^ Riley Hospital Division of Pediatric Hematology Oncology Indianapolis Indiana USA; ^5^ Indiana Cancer Consortium Indianapolis Indiana USA; ^6^ Indiana University Melvin and Bren Simon Comprehensive Cancer Center Indianapolis Indiana USA; ^7^ Indiana University School of Public Health Bloomington Indiana USA; ^8^ Prevention Insights Bloomington Indiana USA; ^9^ Department of Child Health University of Missouri School of Medicine Columbia Missouri USA

**Keywords:** cancer education, cancer prevention, clinical management, screening, survival

## Abstract

**Introduction:**

An estimated 39,010 Indiana residents were diagnosed with cancer in 2021. To address the cancer burden, Project ECHO (Extension Community Healthcare Outcomes) was launched in 2019 in Indiana to build specialty healthcare capacity among non‐specialists. Due to positive outcomes from the pilot year, the Cancer Prevention, Screening, and Survivorship ECHO was implemented for a second year. The purpose of this study was to measure the participation and regional impact of this ECHO.

**Methods:**

ECHO sessions occurred twice monthly from October 2020 to October 2021. Changes were implemented in response to feedback from the pilot year, including making the curriculum more practical for learners and adding accreditation opportunities. Participant information and feedback was extracted from electronic surveys for review.

**Results:**

There were 24 ECHO sessions with 213 unique participants, increased from 140 unique participants in the pilot year. An average of 23.5 individuals attended each session, increased from 15.5 individuals per session. Enrolled participants served in a diverse set of roles and represented 247 zip codes, 30 Indiana counties, and 32 states across the United States, each of which increased from the pilot year.

**Discussion:**

In this second year, this ECHO expanded to reach more participants with increased attendance and a more diverse distribution of roles within healthcare, which may be attributed to feedback‐driven curriculum design. Cancer care is multi‐disciplinary, with health educators, nurses, and administrators, each acting within the cancer care continuum. As a result, this ECHO has been adapted to serve an increasingly broad distribution of professionals.

**Conclusion:**

The second year of the Cancer Prevention, Screening, and Survivorship ECHO displayed increased overall enrollment and participation, greater diversity among participant roles, and a wider reach across Indiana and the United States.

## Graphical abstract

To address the cancer burden, Project ECHO (Extension Community Healthcare Outcomes) was launched in 2019 in Indiana to build specialty healthcare capacity among non‐specialists on topics in cancer prevention, screening, and survivorship care. In this second year, this ECHO reached 213 unique participants in 247 zip codes, 30 Indiana counties, 32 states, and 4 countries. This ECHO expanded to reach more participants with increased attendance and a more diverse distribution of roles within healthcare, which may be attributed to feedback‐driven curriculum design created to be more accessible.

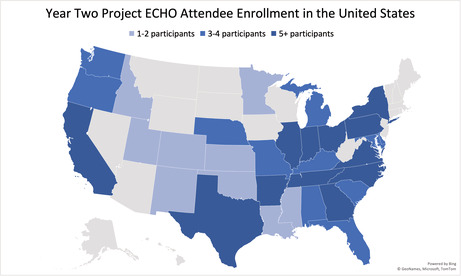

## 1 BACKGROUND

### 1.1 Cancer disparities in Indiana

An estimated 39,010 Indiana residents were diagnosed with cancer in 2021, equating to more than four new cases of cancer diagnosed every hour.^1^ In Indiana, cancer remains the second leading cause of death in adults and children.^1^ The Indiana Cancer Consortium (ICC) aims to reduce cancer morbidity and mortality in Indiana through “coordinated, collective action of its members and the sharing of resources, knowledge, and passion.” The ICC works at each stage of the continuum of care, including primary prevention, early detection, treatment, and survivorship.^2^ The Indiana Department of Health (IDOH) works alongside and provides funding for the ICC to improve cancer care across the State.

### 1.2 Project ECHO

Project ECHO (Extension for Community Healthcare Outcomes) was developed in 2003 in Albuquerque, New Mexico to improve knowledge and competency of care for patients with hepatitis C.^3^ With support from the ECHO Institute at the University of New Mexico, ECHO programs have proliferated nationally and internationally, with other sites focusing on a wide variety of health‐related needs, populations, and disparities, typically to improve care in low‐resource settings or areas where specialty care may be hard to access.^4^, ^5^, ^6^, ^7^, ^8^ In the context of the COVID‐19 pandemic, ECHO programs have continued to fill the gap between specialty care and community providers.^9^, ^10^

**FIGURE 1:**
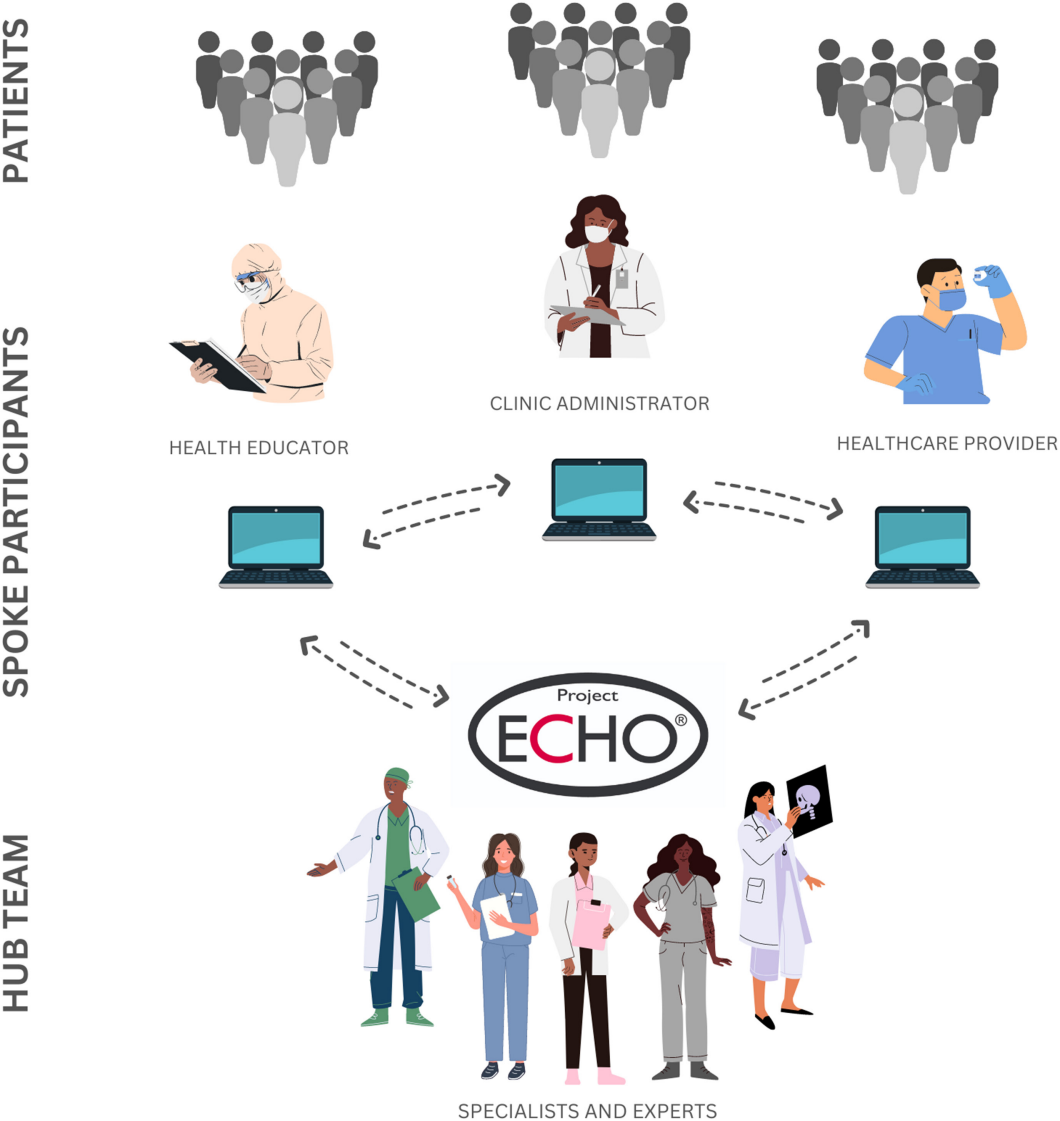
A representation of the educational learning loop where a bidirectional flow of knowledge between care teams can impact an exponentially greater number of patients. Created using Canva with permission from the ECHO Institute.

The Project ECHO model uses tele‐mentoring to provide didactic continuing education and case‐based learning, which allow expert clinicians to mentor other healthcare providers on specialized topics through collaboration and dialogue. Each ECHO program's membership consists of a “hub” team of experts and various “spokes” who have diverse healthcare roles within their communities, as shown in Figure 1. ECHO programs typically meet for 60 to 90‐minute video conferencing sessions at regular intervals (weekly, bi‐weekly, or monthly).^11^

In 2019, the Indiana University Richard M. Fairbanks School of Public Health (FSPH) ECHO Center, the ICC, and the IDOH implemented a Cancer Prevention, Screening, and Survivorship curriculum delivered using the Project ECHO model.^11^, ^12^, ^13^ Although there are more than 3,000 ECHO programs globally, few of those programs focus on cancer care and survivorship.^11^ At the same time, this is an important topic about which to build specialty care capacity. Therefore, this manuscript continues our reporting on the implementation and ongoing functions of the Cancer Prevention, Screening, and Survivorship ECHO in Indiana, with the aim of providing data on participation and engagement that may be useful to other state‐ and national‐level teams seeking to disseminate best‐practice approaches to cancer care and survivorship.

## 2 METHODS

### 2.1 Implementation of the second year of project ECHO

The baseline implementation infrastructure for the Cancer Prevention, Screening, and Survivorship ECHO was described in our pilot manuscript and retained for this second year of operation.^14^ The hub team was recruited from pilot team members, each of whom serves as a specialist or expert in a wide range of oncology topics and geographically represents our catchment area (Indiana). Members of the original hub team were encouraged to recruit new hub team members from their own networks and colleagues to create the second‐year team.

Utilizing the feedback from the surveys and focus groups from the pilot year, several key changes were made to the ECHO.^14^, ^15^ First, the second‐year curriculum of 24 planned ECHO sessions, including didactic sessions with relevant cases (see Table S1), was designed to be more accessible for learners from different educational backgrounds. A voluntary post‐session survey continued to be offered to gather feedback for real‐time quality improvement to adapt the curriculum. Additionally, the second‐year curriculum was adapted based on the perceived needs of the region as determined by the Indiana Cancer Control Plan.^16^ For instance, the plan suggested the need for attention to COVID‐19 care within the cancer continuum, which became the first session of the curriculum (Table S1). Second, an opportunity was added for certified health education specialists (CHES) to obtain continuing education credit by attending the live ECHO sessions. Third, a marketing and communication specialist was hired for the FSPH ECHO Center who recruited participants using social media, including Twitter, LinkedIn, and Facebook.

Finally, an attempt was made to improve attendance and recruitment for the Cancer Prevention, Screening, and Survivorship ECHO by alternating meeting times (12 pm and 4 pm) to accommodate different schedules. After several months of utilizing this schedule, feedback from stakeholders noted participation was limited at 4 pm, particularly for clinicians, and the original timing (12 pm for all sessions) was restored.

### 2.2 Evaluating ECHO participation and engagement

This study was designed as a retrospective review of the ECHO participant database and a voluntary participant survey. The primary aim was to understand how participation and feedback at ECHO sessions in the second year of implementation compared to the first year. To participate in the Cancer Prevention, Screening, and Survivorship ECHO, individuals registered through an online database which stored data in RedCap.^11^ All information used to assess this second year of the program was extracted from RedCap, including the number of participants attending each session, participant location, and occupation. The data were analyzed using basic statistics and compared to the data from the pilot year.

Evaluative efforts during the pilot year included voluntary surveys administered at the end of each ECHO session, which provided feedback to improve content and delivery, as well as focus groups and interviews with five different ECHO programs offered through the FSPH ECHO Center, including the Cancer Prevention, Screening, and Survivorship ECHO.^15^ During the second year, the voluntary surveys were again administered at the end of each ECHO session.

## 3 RESULTS

**FIGURE 2:**
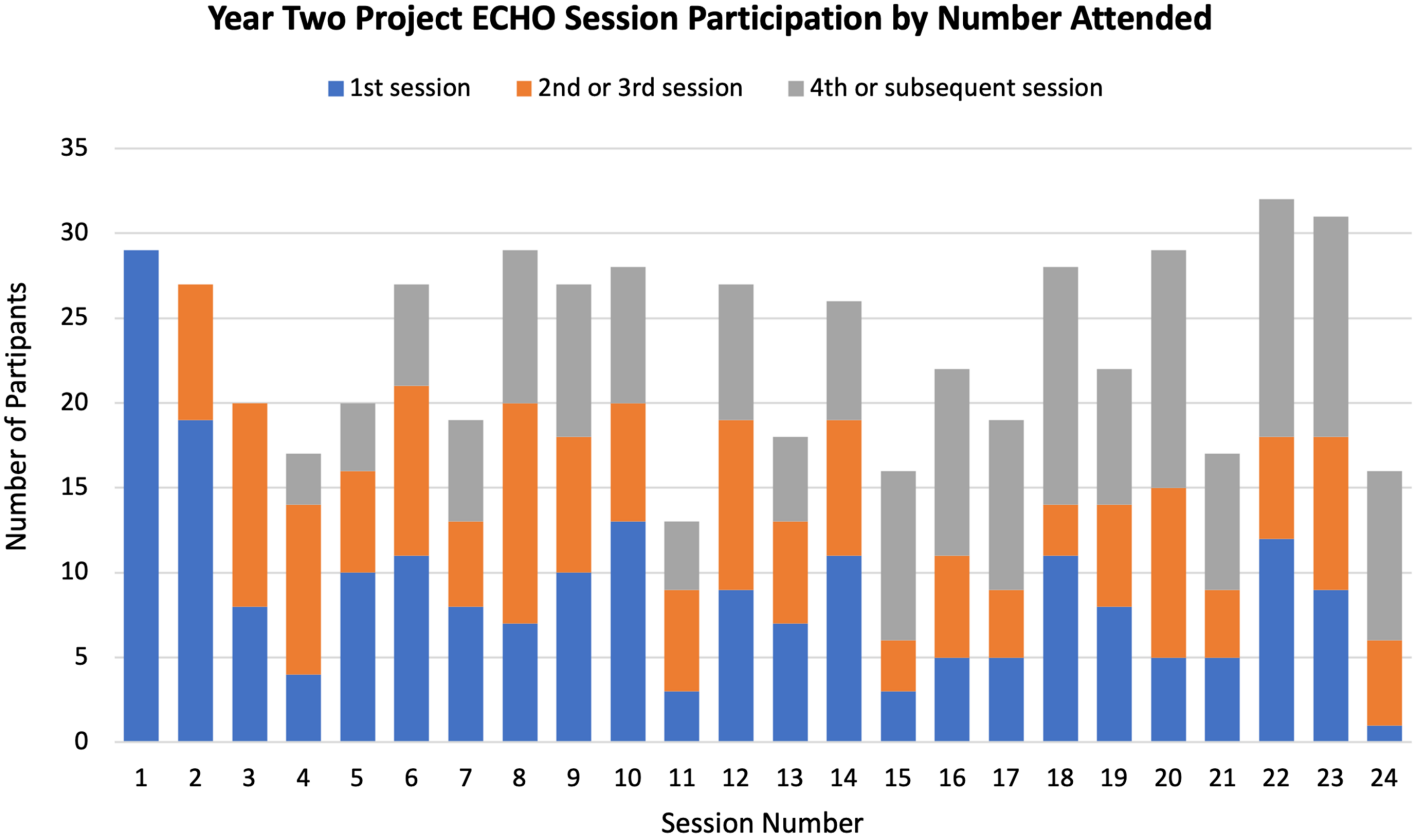
Graphic representation of the spoke site attendance at each of the 24 sessions during the year with segments representing recurrent participation.

A total of 484 people registered to participate, with 213 unique participants attending at least one session throughout the year. This represented an increase in overall engagement with the program compared to the pilot year, which saw 270 registrants and 140 unique participants, though the ratios of registrants to participants were somewhat similar (44% for Year Two, and 52% for Year One). Mean spoke attendance in the second year of the program was higher than in the first year, as shown in Figure 2. Each of the 24 sessions had an average attendance of 23.5 spoke participants, an increase from the mean of 15.5 spoke participants in the pilot year. An average of 10 hub team members attended each session.

**FIGURE 3:**
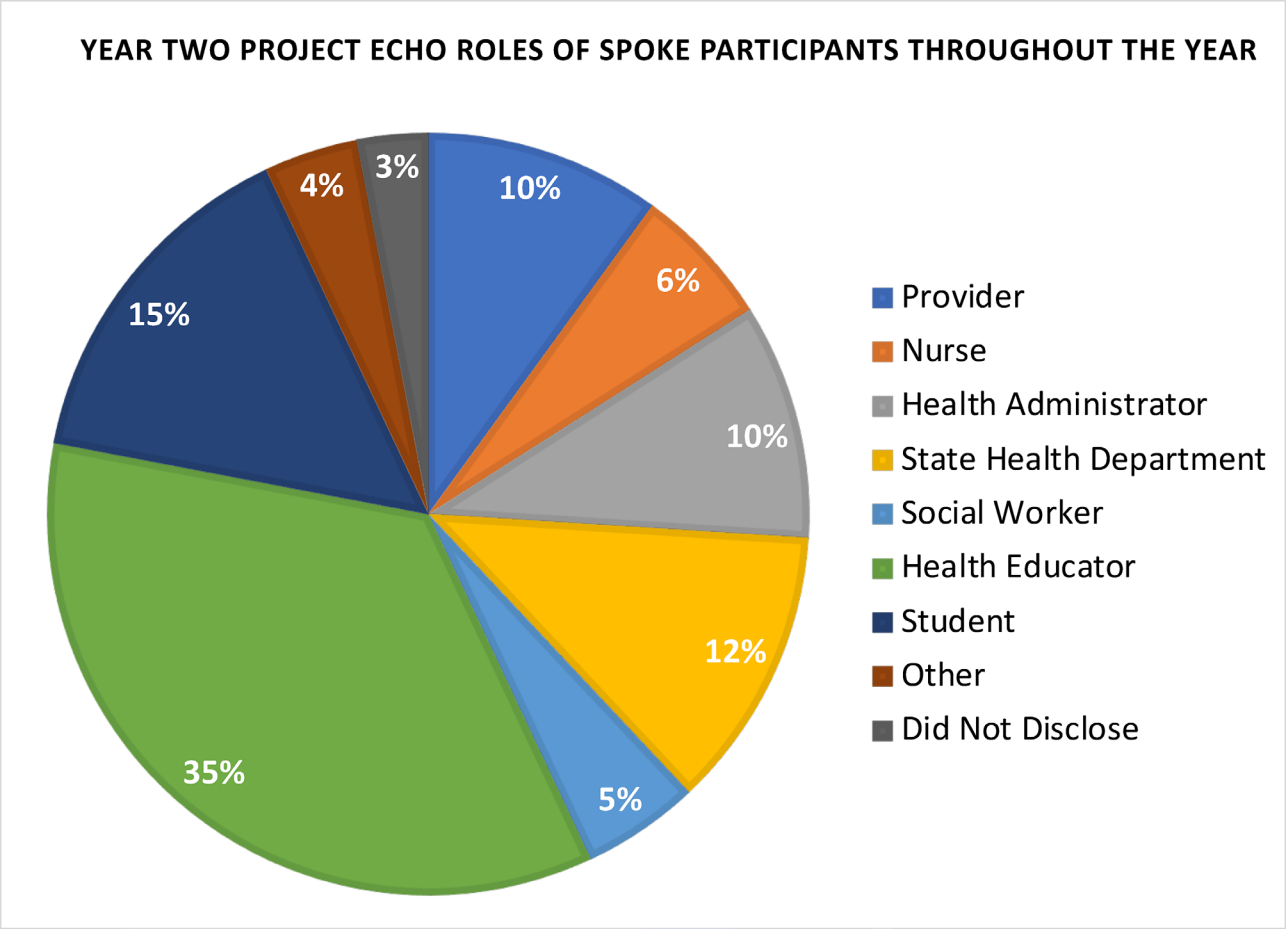
Graphic representation of the total distribution of roles for spoke participants at all ECHO sessions.

Participants who attended ECHO sessions came from various employment settings (see Figure 3), and included 35% health educators, 15% students, 12% state health department employees, 10% providers (physicians, nurse practitioners, physician assistants), 6% nurses, 5% social workers, and 3% who did not disclose. The remaining 4% were considered “other” which included cancer prevention specialists, university professors, pharmacists, certified trauma responders, and physical therapists. Each of these comprised <1% of the total. In the pilot year, notable participant roles included providers (17%), health educators (17%), registered nurses (10%), and state health department employees (7%).

**FIGURE 4:**
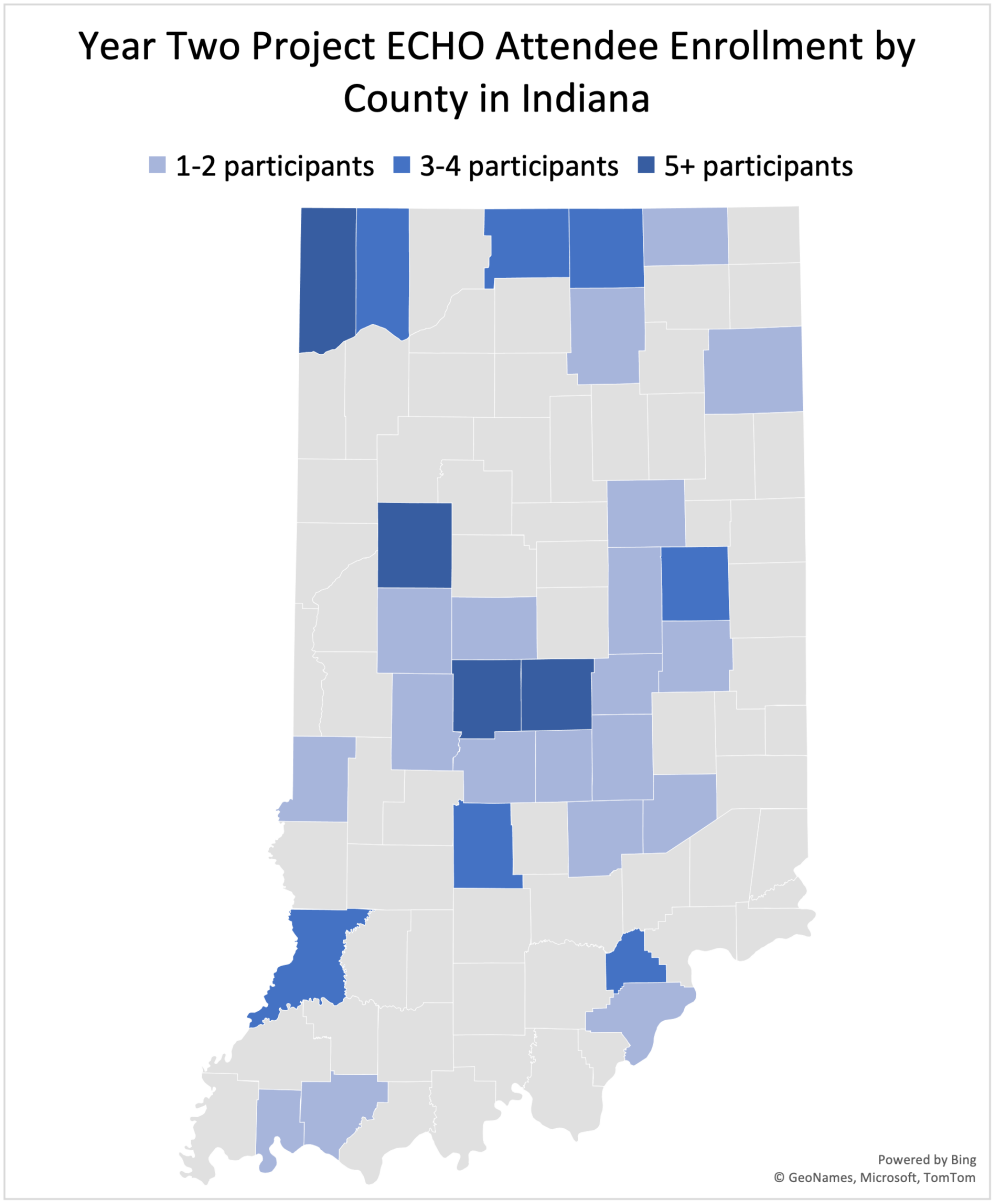
State map of Indiana where highlighted counties represent those enrolled in Cancer ECHO. Map generated using Microsoft Excel.

**FIGURE 5:**
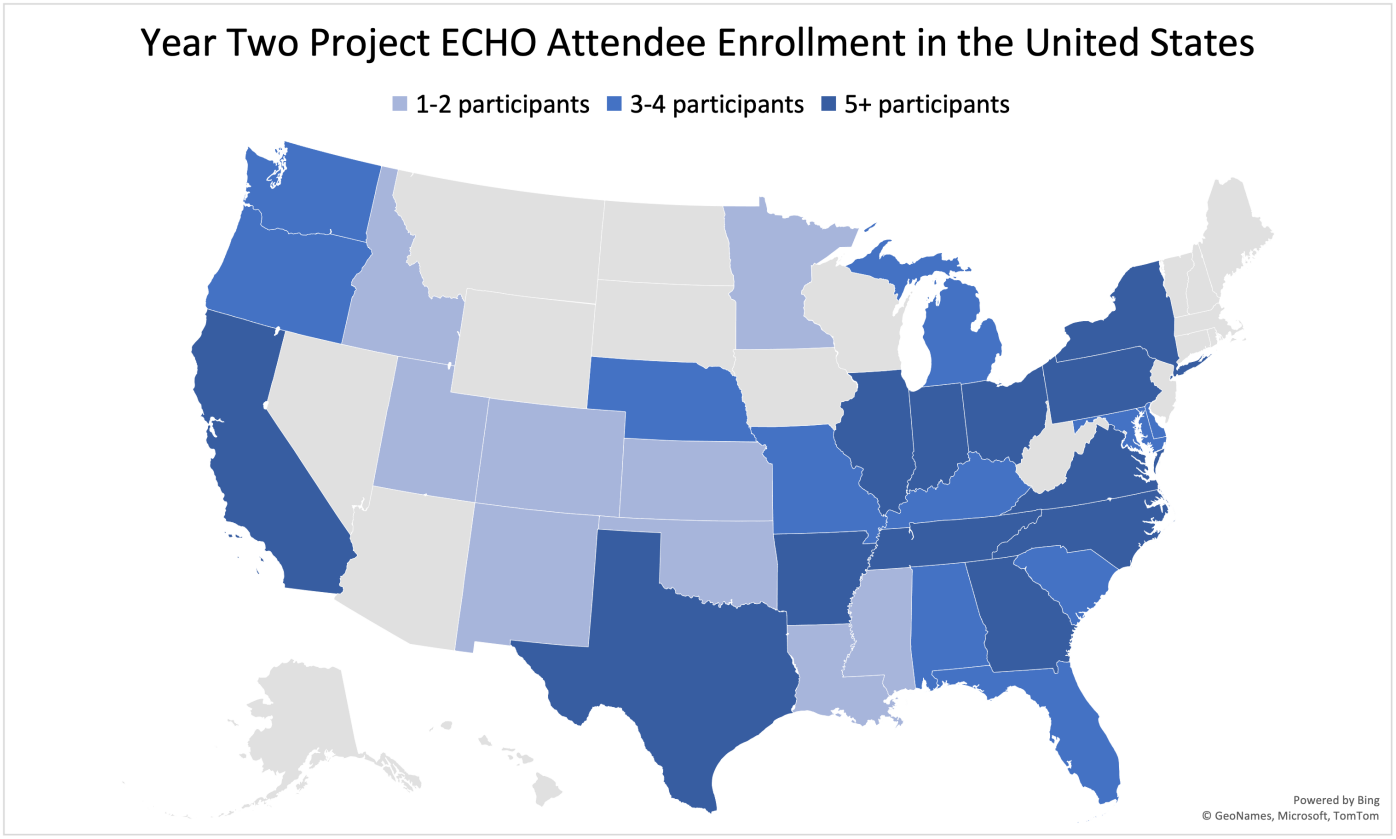
Map of the United States where highlighted states represent those enrolled in Cancer ECHO. Map generated using Microsoft Excel.

Enrolled participants were substantively more geographically dispersed, representing 247 different zip codes, compared to 75 different zip codes during the pilot year. Within the state of Indiana, 30 different counties were represented, increased from 19 counties previously (Figure 4). In the United States, a total of 32 states were represented (Figure 5), which increased from 22 states the year prior. There was a continued international presence as well, with participants from Germany and the Philippines. This was similar to the pilot year, where spoke participants attended from Kenya, Nigeria, and Lebanon.

During the pilot year, 28 spoke participants were considered consistent attendees (defined as attending at least four sessions). Using that same metric, in the second year of implementation, there were 45 consistent attendees. From the pilot year to the second year, 16 consistent attendees returned, attending at least one session. Out of those 16 consistent attendees in the pilot year, 12 were consistent attendees again in the second year and one became a hub team member.

After each session, participants were invited to complete a brief survey. In total, 98 post‐session surveys were completed (17% of the possible surveys that could have been completed). Across the completed surveys, 74% stated that the objectives of the session were met “extremely well” and 80% rated the quality of the didactic session as “excellent,” increased from 72% during the pilot year. In both the first and the second year, no participants reported the objectives were “not met well” or were only “slightly met well,” and no responders felt the session was of “terrible” or “poor” quality.

## 4 DISCUSSION

In this second year, the Cancer Prevention, Screening, and Survivorship ECHO successfully expanded to reach more participants with increased attendance and broadening representation across Indiana and the United States. Compared to the pilot year, participants also came from a more diverse distribution of educational backgrounds and roles within healthcare. As the program becomes more established over time, some growth is to be expected, which may also be reflected in increased participant diversity. The breadth of attendee practice locations may also allow inference for the continued need of such programs since our program is nominally an “Indiana program” but has considerably wider reach.

At the same time, we note that extensive efforts were also made to respond to participant feedback to meet attendees' needs as closely as possible, which may have been associated with this growth. Furthermore, we suspect, but cannot definitively claim, that the program's substantial growth was associated with our dedicated effort to increase participation by hiring a marketing coordinator to reach potential participants using social media.

It is also important to view the shift in our ECHO program within the broader context of Project ECHO. When it was first designed at the University of New Mexico, Project ECHO was mainly directed toward primary care providers and those who work directly with patients with chronic illnesses such as hepatitis C and HIV.^3^, ^11^ Cancer control arguably represents a much broader challenge as there are extensive points along the cancer continuum where patients interact with a myriad of health professionals, not just primary care providers. This is reflected in the broad distribution of providers who attended our cancer‐focused ECHO program, each of whom represented roles involved in the continuum of care, such as educators, therapists, epidemiologists, and program managers. This ECHO evolved to meet the expectations of this broader group, but at the same time, has not yet fostered the anticipated level of engagement from primary care providers.

Undoubtedly, the COVID‐19 pandemic had an impact on the Cancer Prevention, Screening, and Survivorship ECHO in this second year of implementation. Globally, the pandemic saw a shift in medical education to virtual platforms to provide learning opportunities to individuals outside of their typical avenues.^17^, ^18^ In these new formats, individuals from out of state or across the country could be included for the first time. During the pandemic, it appears that learners were more likely to explore educational opportunities outside of their routine methods, which were in turn, adapting to include a more diverse set of learners.^18^ This may contribute to not only the increase in users, but also the increase in consistent users from the pilot year. As individuals feel more comfortable and engaged in the material, they may be more likely to return for another session. The ECHO model was originally designed to be based around collaborative dialogue as the basis for education, with the slogan “all teach, all learn.”^11^ With increased consistency and more familiarity with this ECHO, participants have more opportunities to develop comfortability and openness to dialogue.

There were several limitations to this study. The use of survey tools carries inherent biases, such as nonresponse bias, since the feedback we received was from a select subgroup of participants. Additionally, the ECHO platform for documenting participation had limitations. For example, if a participant joined the ECHO session by calling in on a mobile device, rather than using the Zoom link, we were unable to match them with their ECHO profile. As a result, those who called in could not be identified for their role or geographical location. In addition, funding for ECHO remains a barrier. As of now, the hub team is volunteer, requiring no salary support. Had members of the hub team or facilitators of case discussions needed salary support, the cost to sustain ECHO would have been much more challenging.

In future studies of the Cancer Prevention, Screening, and Survivorship ECHO, we plan several improvements. First, our team plans to place a greater emphasis on screening rates in regions with known ECHO participants. This will allow the team to compare these rates to state and county averages to better capture the impact of Project ECHO at the patient level. To better understand which outreach efforts are most impactful, two questions were added to the registration form that ask how the individual learned about ECHO. Additionally, focus groups will be utilized to identify key reasons as to why some participants only attended one session; we note that a small study of “drop out” participants from the 2018 Indiana Opioid Use Disorder ECHO found that practical constraints (such as availability) tended to be a primary reason.^19^ Finally, more information will be gathered on participants, such as age, to better understand participant demographics. This could help identify any potential barriers some attendees may encounter to accessing this ECHO.

## 5 CONCLUSION

The Project ECHO platform is a well‐established model with the capacity to virtually reach medically isolated communities beyond geographical borders to better empower medical and other health‐related professionals to care for patients throughout the cancer continuum. The second year of the Cancer Prevention, Screening, and Survivorship ECHO displayed increased enrollment and participation, greater diversity among participant roles, and a wider reach across Indiana and the United States. This pattern and distribution of growth highlight both the potential broad reaching impact of tele‐mentoring education in cancer care and the requirement for ECHO hubs to remain flexible and adapt to the needs of stakeholders.

## AUTHOR CONTRIBUTIONS

**Mary Ann Etling:** Conceptualization (equal); data curation (lead); formal analysis (lead); investigation (supporting); writing – original draft (lead); writing – review and editing (lead). **Terry A Vik:** Conceptualization (equal); investigation (supporting); project administration (equal); supervision (equal); writing – review and editing (equal). **Andrea Janota:** Conceptualization (equal); supervision (equal); writing – review and editing (equal). **Kaley L Liang:** Conceptualization (equal); methodology (equal); resources (equal); software (equal); writing – review and editing (equal). **Caroline L Kryder‐Reid:** Conceptualization (equal); methodology (equal); resources (equal); software (equal); writing – review and editing (equal). **Mary Robertson:** Conceptualization (equal); methodology (equal); writing – review and editing (equal). **Caitlin Scanlon:** Conceptualization (equal); methodology (equal); writing – review and editing (equal). **Anyé Carson:** Conceptualization (equal); methodology (equal); writing – review and editing (equal). **Jon Agley:** Conceptualization (equal); validation (lead); writing – review and editing (equal). **Tyler S Severance:** Conceptualization (lead); data curation (equal); formal analysis (equal); investigation (lead); methodology (equal); project administration (lead); supervision (lead); writing – original draft (equal); writing – review and editing (equal).

## FUNDING INFORMATION

The Cancer Prevention, Screening, and Survivorship ECHO program described received funding or in‐kind support from the Indiana Immunization Coalition, Indiana Department of Health, Riley Hospital for Children, the American Cancer Society, Indiana Cancer Consortium, Indiana Clinical and Translational Sciences Institute, the Richard M. Fairbanks School of Public Health, and the Indiana University Addictions Grand Challenge. This work was supported in part by the IU Simon Comprehensive Cancer Center William J. Wright Scholarship awarded to MAE.

## ETHICAL APPROVAL STATEMENT

This study was approved by the Indiana University Institutional Review Board.

## Supporting information


Table S1

## Data Availability

The data that supports the findings of this study is available from the corresponding author upon reasonable request.
