# Supplementary material for: The continuing evolution of a cancer prevention, screening, and survivorship ECHO: A second year of implementation
Source: Cancer Med. 2022 Dec 12;12(6):7398–405. doi: 10.1002/cam4.5441 (PMC10067045; doi:10.1002/cam4.5441)
Supplement: Supplementary file 1 — Table S1 [file CAM4-12-7398-s001.docx]

| **Session Number** | **Date** | **Session Topic** |
| --- | --- | --- |
| 1 | 10/20/20 | PCP and Cancer Survivorship |
| 2 | 11/3/20 | Lung Cancer and Vaping Approaches to Screening Based on Age Group, Disparity Rates of Survivors |
| 3 | 11/17/20 | Motivational Interviewing in Smoking Cessation |
| 4 | 12/1/20 | Navigating Scholarly Resources |
| 5 | 12/15/20 | IDH Cancer Programs Coverage on Comprehensive Cancer Program |
| 6 | 1/5/21 | IDOH BCCP Program |
| 7 | 1/19/21 | Flu/Fit Campaign |
| 8 | 2/2/21 | Indiana Cancer Control Plan |
| 9 | 2/16/21 | HPV Updates |
| 10 | 3/2/21 | Predisposed Factors for Cancer Part 1 |
| 11 | 3/16/21 | Patient-Centered Language |
| 12 | 4/6/21 | Predisposed Factors for Cancer Part 2 |
| 13 | 4/20/21 | PCP and Cancer Survivorship |
| 14 | 5/4/21 | Smoking with a Cancer Diagnosis |
| 15 | 5/18/21 | Nutrition |
| 16 | 6/1/21 | COVID-19 Care |
| 17 | 6/15/21 | Cancer Health Disparities for People with Disabilities |
| 18 | 7/6/21 | Indiana's Survivorship Data and IDOH Learning Collaborative |
| 19 | 7/20/21 | State Tobacco Efforts |
| 20 | 8/3/21 | HPV Counseling |
| 21 | 8/17/21 | Cancer Screening, Prevention, and Treatment Among People Living with HIV/AIDS |
| 22 | 9/7/21 | Trauma-Based Care and QI for HPV Screening |
| 23 | 9/21/21 | Cancer Advocacy |
| 24 | 10/5/21 | Genetic Counseling |

**Table S1.** Didactic topics for each of the sessions of the ECHO curriculum.
